# CENPM as a biomarker and therapeutic target for lymph node metastasis in thyroid carcinoma

**DOI:** 10.3389/fgene.2026.1875148

**Published:** 2026-07-22

**Authors:** Ligong Zhang, Bing Wang, Mingliang Zhang

**Affiliations:** Department of Surgical Oncology, The First Affiliated Hospital of Bengbu Medical University, Bengbu, Anhui, China

**Keywords:** CENPM, generalized additive model, lymph node metastasis, single-cell analysis, thyroid carcinoma

## Abstract

**Background:**

Lymph node metastasis (LNM) is a key prognostic determinant in thyroid carcinoma (THCA), yet molecular markers capturing intrinsic metastatic potential are limited.

**Methods:**

Generalized additive models were applied to TCGA-THCA data to screen for genes with diametrically opposite expression–tumor diameter relationships between N0 and N1 patients. CENPM was subsequently validated in independent transcriptomic cohorts, spatial transcriptomics, and immunohistochemistry. Single-cell transcriptomics, *in silico* knockout, drug repositioning, and molecular docking were employed to dissect its immunological roles and therapeutic relevance.

**Results:**

CENPM expression increased with tumor diameter in N0 but decreased in N1, and high CENPM was associated with poorer disease-free survival. CENPM was predominantly enriched in CD8^+^ naïve and effector T cells, particularly in anaplastic carcinoma. Virtual knockout predicted downstream transcriptional changes associated with lymphocyte activation, translational machinery, and immune effector pathways. Drug repositioning identified filgotinib as a candidate to reverse the CENPM-high signature, with stable CENPM–filgotinib binding confirmed by docking and molecular dynamics.

**Conclusion:**

CENPM shows diametrically opposite expression–diameter relationships between N0 and N1 patients, suggesting a shift in biological behavior upon nodal involvement, with potential therapeutic relevance.

## 1 Introduction

Thyroid carcinoma (THCA) represents the most prevalent endocrine malignancy, with GLOBOCAN 2022 estimating 821,214 new cases and 47,507 deaths worldwide (ASIR: 13.60/100,000 in women, 4.60/100,000 in men) (Lyu et al., 2024). Notably, 64.63% of cases occur in individuals under 55 years, whereas 82.99% of mortality concentrates in those aged 55 and above, highlighting the striking discord between incidence and lethality (Lyu et al., 2024).

The histological spectrum of THCA demonstrates that lymph node metastasis (LNM) constitutes a critical discriminator of metastatic virulence rather than mere anatomical dissemination. Anaplastic thyroid carcinoma (ATC), accounting for merely 1%–2% of cases yet causing over half of thyroid cancer mortality, exhibits cervical nodal involvement in 40%–70.69% of patients at diagnosis (Rao and Smallridge, 2023). Conversely, papillary thyroid carcinoma (PTC), comprising over 85% of cases, demonstrates a biological dichotomy: while micrometastatic disease is detected in up to 90% of central neck (Level VI) specimens upon rigorous histopathological scrutiny, clinically evident metastases are identified in only 20%–50% of patients, with minimal prognostic impact (Agarwal et al., 2012). This contrast underscores that the *presence* of LNM inadequately captures metastatic potential. The anatomical distribution further stratifies risk: lateral compartment (Levels I–V) involvement occurs in 6.8%–37.5% of cases, whereas Level VII (superior mediastinal) metastasis, though less frequent, confers the poorest cancer-specific survival (HR 5.67 versus N0) (Ouyang et al., 2025). Prognostically, extranodal extension (ENE) and lymph node ratio (LNR) ≥0.42 emerge as critical determinants of regional recurrence and disease-specific mortality, with N1b disease and >3 metastatic central nodes significantly compromising 5-year recurrence-free survival (Xu et al., 2022).

Current diagnostic and therapeutic paradigms remain fundamentally constrained by their reliance on anatomical staging. Preoperative ultrasonography and computed tomography exhibit limited sensitivity for subclinical central compartment involvement, forcing a dichotomous choice between understaging with therapeutic omission or overstaging with unnecessary surgical morbidity (Guo et al., 2025). The ATA guidelines recommend therapeutic lateral neck dissection only for clinically evident disease, explicitly discouraging prophylactic dissection due to risks of permanent hypoparathyroidism and recurrent laryngeal nerve injury without proven survival benefits; however, this conservatism potentially compromises locoregional control (Guo et al., 2025). Molecular staging currently relies on BRAF V600E and TERT promoter mutations, which, while associated with aggressive phenotypes, lack sufficient predictive precision for individualized nodal risk stratification (Guo et al., 2024). These limitations underscore the imperative necessity for novel biomarkers capable of quantifying intrinsic “lymph node metastatic potential”—defined as the inherent biological propensity for lymphatic dissemination—thereby enabling a paradigm shift from anatomical documentation to molecular risk assessment.

The Cancer Genome Atlas (TCGA) provides comprehensive transcriptomic profiling of 507 primary THCA samples (N0, n = 220; N1, n = 218) with annotated tumor dimensions, offering an unprecedented resource to dissect these molecular heterogeneities. By applying generalized additive models (GAM) to elucidate non-linear relationships between gene expression and tumor diameter within distinct nodal cohorts, this study aims to identify genes whose expression patterns correlate with metastatic potential (Ravindra et al., 2019). We hypothesize that genes exhibiting diametrically opposed expression-diameter relationships between N0 and N1 cohorts, or N1-specific associations with small-diameter tumors indicating high metastatic propensity independent of tumor bulk, may constitute robust molecular surrogates for lymph node metastatic potential.

The identification of such biomarkers would address critical unmet clinical needs: enabling preoperative risk stratification to guide surgical extent, revealing therapeutic targets specific to the metastatic cascade, and refining postoperative surveillance protocols through biological phenotyping.

## 2 Materials and methods

### 2.1 Data source and preprocessing

RNA-sequencing data (TPM values) and corresponding clinicopathological information for THCA were downloaded from TCGA. Primary tumor samples were extracted and divided into two groups based on LNM status: lymph node-negative (N0, n = 220) and lymph node-positive (N1, n = 218). The maximum diameter of the primary tumor (Diameter, in cm) was recorded for each sample. Genes with extremely low expression variance (<0.01 across all samples) were removed. Differentially expressed genes (DEGs) between tumor and normal tissues in the TCGA-THCA cohort were obtained from the GEPIA2 database (http://gepia2.cancer-pku.cn), with screening criteria set at |Log_2_FC| > 1 and q-value < 0.05, resulting in 4,099 differentially expressed genes.

## 3 Generalized additive model (GAM) fitting

To flexibly capture potential nonlinear relationships between gene expression and tumor diameter, we employed a GAM (Ravindra et al., 2019; Mullah et al., 2019; Cui et al., 2019; Feng, 2022; Souza Silva et al., 2020; Qin et al., 2024; Liu et al., 2022a). As a non-parametric extension of generalized linear models, GAM automatically learns nonlinear trends through smooth functions while preserving interpretability. For each gene, independent models were fitted in the N0 and N1 groups with the following specification:

$$\text{Expression}=\beta 0+s\left(\text{Diameter}\right)+\varepsilon$$

where *β*0 is the intercept; *s* is a smooth function of tumor diameter, implemented using thin plate regression splines as basis functions. Thin plate splines incorporate a natural penalty that balances goodness-of-fit and smoothness, and they avoid the need for manual knot placement; *ε* is the random error term, assumed to follow a normal distribution. To prevent excessive curvature (overfitting), the maximum basis dimension of the smooth term was limited to k = 5, i.e., the maximum effective degrees of freedom (edf) ≤ 4. This choice was based on the sample size (approximately 220 per group) and the expected complexity of nonlinearity, allowing meaningful curvature while avoiding noise fitting. Model parameters were estimated using restricted maximum likelihood (REML). REML automatically selects optimal smoothing parameters while providing unbiased estimates of fixed effects; it is more stable than generalized cross-validation (GCV), especially with moderate sample sizes.

After model fitting, we evaluated effective degrees of freedom (edf), F-statistic with associated p-value, and deviance explained to assess model quality. Effective degrees of freedom indicate the degree of curvature, where an edf of 1 denotes a purely linear relationship while edf > 1 suggests nonlinearity. The F-statistic and associated p-value test whether the smooth term is significantly different from zero (null hypothesis: the smooth term is constant). Deviance explained, analogous to *R*2 in linear regression, represents the proportion of expression variance accounted for by tumor diameter. All GAM analyses were performed using the mgcv package in R. Cases with convergence failure or singular fits were recorded as missing and excluded from subsequent analyses.

## 4 Pre-filtering of genes

To avoid overfitting driven by extreme expression values or highly discrete distributions, we applied the following pre-filters to each group before GAM fitting: number of unique expression values must be at least 5; proportion of zero expression values must not exceed 50%; ratio of maximum to median expression must not exceed 50; and coefficient of variation (CV) must not exceed 3. Genes failing any of these criteria were excluded from further GAM fitting and screening.

## 5 Screening strategy for genes associated with lymph node metastatic potential

Based on clinical observations: among patients without LNM (N0), the larger the primary tumor diameter, the higher the potential risk of future LNM, indicating a relatively higher intrinsic lymph node metastatic potential (though not yet metastasized) (Machens et al., 2005; Verburg et al., 2009; Chen et al., 2025; Luo et al., 2024; Walczyk et al., 2025). In contrast, among patients with LNM (N1), a smaller primary tumor diameter accompanied by nodal spread suggests extremely high lymph node metastatic potential (small diameter with metastatic capability). Accordingly, we hypothesized that genes associated with lymph node metastatic potential might show opposite expression-diameter relationships between the two groups.

Screening pattern (opposite effects): Positive association in N0 (large diameter with high expression, consistent with a relatively higher metastatic potential trend) and negative association in N1 (small diameter with high expression, consistent with extremely high metastatic potential). The GAM smooth term p-value < 0.05 in both groups.

The core biological significance of this pattern lies in: genes upregulated with increasing tumor size in the N0 group reflect an activated metastatic preparation state during tumor growth, whereas in the N1 group, high expression is observed even in small-diameter tumors, suggesting their capacity to drive early metastasis. This “opposing” expression pattern may represent true metastatic potential markers rather than mere tumor burden-related genes.

## 6 Robustness criteria

For genes meeting the above pattern, we further applied the following robustness criteria to exclude overfitting: effective degrees of freedom (edf) must be less than 3.0, and deviance explained (dev_expl) must be less than 0.3. To reliably determine the direction of association, we calculated the predicted difference Δ as prediction(Diameter_max_)−prediction(Diameter_min_), where Δ > 0 indicates a positive association and Δ < 0 indicates a negative association (Ravindra et al., 2019; Mullah et al., 2019; Cui et al., 2019; Feng, 2022; Souza Silva et al., 2020; Qin et al., 2024; Liu et al., 2022a).

## 7 Single-cell RNA sequencing data processing

The scRNA-seq dataset (GSE193581) was downloaded from the NCBI Gene Expression Omnibus (GEO) database. This dataset contains 23 thyroid tissue samples, including 10 ATC, 7 PTC, and 6 adjacent normal thyroid tissues (Ning et al., 2025). Raw gene expression matrices were processed using the Seurat R package (Wang et al., 2024; Slovin et al., 2021; Zhang et al., 2025). For each sample, cells with fewer than 200 detected genes, genes detected in fewer than 3 cells, and cells with >10% mitochondrial gene expression were excluded to ensure data quality. The filtered data were then log-normalized, and the top 2,000 highly variable genes (HVGs) were identified using the vst method. Principal component analysis (PCA) was performed on the scaled HVG matrix, and the first 30 principal components (PCs) were selected for uniform manifold approximation and projection (UMAP) dimensionality reduction. Graph-based clustering was conducted at a resolution of 0.8, and cell type identities were assigned by canonical marker gene expression and cross-referencing the original publication annotations. T-cell subpopulations (including CD8^+^ Naive, CD8^+^ Effector, and other subsets) were subset and re-clustered for downstream CENPM expression analysis.

## 8 *In silico* gene knockout using scTenifoldKnk

To predict the transcriptional consequences of CENPM knockdown in specific T-cell subsets, we employed the scTenifoldKnk R package, a virtual knockout framework based on single-cell gene regulatory network (scGRN) perturbation (Osorio et al., 2022; Liu et al., 2026; Feng et al., 2026). Briefly, scTenifoldKnk constructs cell-type-specific gene co-expression networks via 10-fold cross-validation from the input single-cell expression matrix. It then performs *in silico* knockout of the target gene (CENPM) by setting its expression to zero and propagating this perturbation through the network using tensor decomposition (Osorio et al., 2022). The resulting differentially affected genes were ranked by Mann–Whitney U test P values and effect sizes. Genes with P < 0.05 and consistent directional changes across network folds were considered significantly altered by CENPM knockout. The top 30 most significantly affected genes were visualized as bar plots colored by P-value gradient.

## 9 Drug repositioning analysis using DrugReflector

For the LNM (N1) subgroup, samples were stratified into high- and low-CENPM expression groups based on the median expression level. DEGs between the two groups were identified using the limma package, yielding log_2_ fold change (logFC) and corresponding P values for each gene. To construct a genome-wide expression signature, all genes were ranked in descending order of logFC, generating a complete ranked list. This list was encapsulated into an h5ad file as required by the DrugReflector platform, with the logFC of each gene serving as its effect size (Chen et al., 2026; Ma, 2026).

DrugReflector leverages the LINCS L1000 database of drug-perturbed expression profiles to identify small-molecule compounds that may reverse the CENPM-high transcriptional signature (Subramanian et al., 2017). This is achieved by calculating the cosine similarity (or CMap connectivity score) between the input signature and drug-induced gene expression changes across various cell lines. A negative correlation (i.e., an inverse relationship between the drug effect and the input signature) suggests therapeutic potential. The analysis outputs a rank, a logit score, and a corresponding probability for each drug. A higher logit score and a lower rank indicate stronger negative correlation with the CENPM-high expression profile. In this study, the top 10 compounds with the highest probability were selected as candidates for subsequent literature validation and molecular docking.

## 10 Molecular docking and molecular dynamics simulation

The two-dimensional structures of small-molecule ligands were retrieved from PubChem and saved in mol2 format. The high-resolution crystal structure of the target protein was obtained from the RCSB Protein Data Bank (Kim et al., 2021; Goodsell and Burley, 2020).

Molecular docking calculations were performed using the CB-Dock2 server (https://cadd.labshare.cn/cb-dock2/), an improved blind docking tool that integrates cavity detection, docking and homologous template fitting (Liu et al., 2022b). The server was run with default parameters. For a given protein–ligand pair, the server first searches its built-in protein-ligand complex database for template ligands with high topological similarity (FP2 ≥ 0.4). When such templates are found, it calculates the sequence identity between the query protein and the proteins complexed with the selected template ligands; complexes with >40% sequence identity and pocket RMSD ≤ 4 Å are retained for template-based cavity detection and docking. For template-based blind docking, the server employs FitDock, an in-house developed method that fits an initial conformation to the template using a hierarchical multi-feature alignment approach, explores possible conformations, and finally outputs refined docking poses. For template-independent blind docking, the latest version (1.2.0) of AutoDock Vina is used. The template database used is BioLiP2 (version 2025.04.23). Interaction patterns were visualized with LigPlus (2D) and PyMOL (3D).

Molecular dynamics (MD) simulations were performed with GROMACS 2022.2 (Kieninger and Keller, 2022). The protein was parameterized using AMBER14SB via pdb2gmx (Huai et al., 2021). Ligand topology was built with sobtop_1.0 (dev3.1) under GAFF2, assigning RESP charges (Wang et al., 2004). The complex was solvated in a cubic TIP3P box with at least 1.0 nm margin, and 0.15 M NaCl was added by gmx genion for charge neutralization. Electrostatics were treated with PME (1.0 nm cutoff), and bonds constrained with LINCS. Energy minimization consisted of stepwise relaxation (solvent → ions → whole system) using 3,000 steepest descent and 2,000 conjugate gradient steps. Production runs of 100 ns were conducted under NPT at 310 K (Nosé–Hoover, τT = 0.1 ps) and 1 bar (Parrinello–Rahman, τP = 2.0 ps) with a 2 fs time step. Stability and structural properties were assessed by RMSD, Cα RMSF, hydrogen bond count, Rg, SASA, and free energy landscape using gmx rmsd, gmx rmsf, gmx hbond, gmx gyrate, gmx sasa, and gmx sham, respectively.

## 11 Immunohistochemistry image densitometric quantification

Whole-slide digital images of CENPM immunohistochemically stained sections were subjected to Average Optical Density (AOD) quantification using a customized Python (v3.12)-based image analysis pipeline. For each section, five non-overlapping Regions of Interest (ROIs; 280 × 280 pixels) were randomly selected within the tissue area. RGB images were deconvolved into Hematoxylin, 3,3′-diaminobenzidine (DAB), and residual channels using the color deconvolution algorithm based on the stain matrix established by Ruifrok and Johnston (2001). In the DAB channel, white background regions were first excluded using saturation-brightness thresholds in the HSV color space. The background baseline was then estimated as the 10th percentile of DAB optical density (OD) values within the tissue-masked region, and the positive staining threshold was set at the background baseline plus 0.05 OD units (Ruifrok et al., 2003). AOD was defined as the mean OD value of positively stained pixels (DAB OD exceeding the threshold). The final AOD value for each section was calculated as the mean of its five randomly selected ROIs. To minimize inter-batch staining variation, Min-Max normalization was applied to linearly transform the mean AOD values of all sections into the 0–1 scale using the formula: Normalized AOD = (AOD−AOD_min) / (AOD_max−AOD_min), where AOD_min and AOD_max represent the minimum and maximum mean AOD values across the entire batch, respectively. The primary antibody against CENPM (rabbit polyclonal, catalog number PA5-50777, Thermo Fisher Scientific) was selected. The specificity and optimal dilution (1:100) were confirmed in pilot experiments using FFPE thyroid tissue sections, where antigen retrieval was optimized using citrate buffer pH 6.0 with high-pressure heating.

## 12 Result

### 12.1 Identification of CENPM as showing opposite expression–diameter patterns between N0 and N1 patients

**FIGURE 1:**
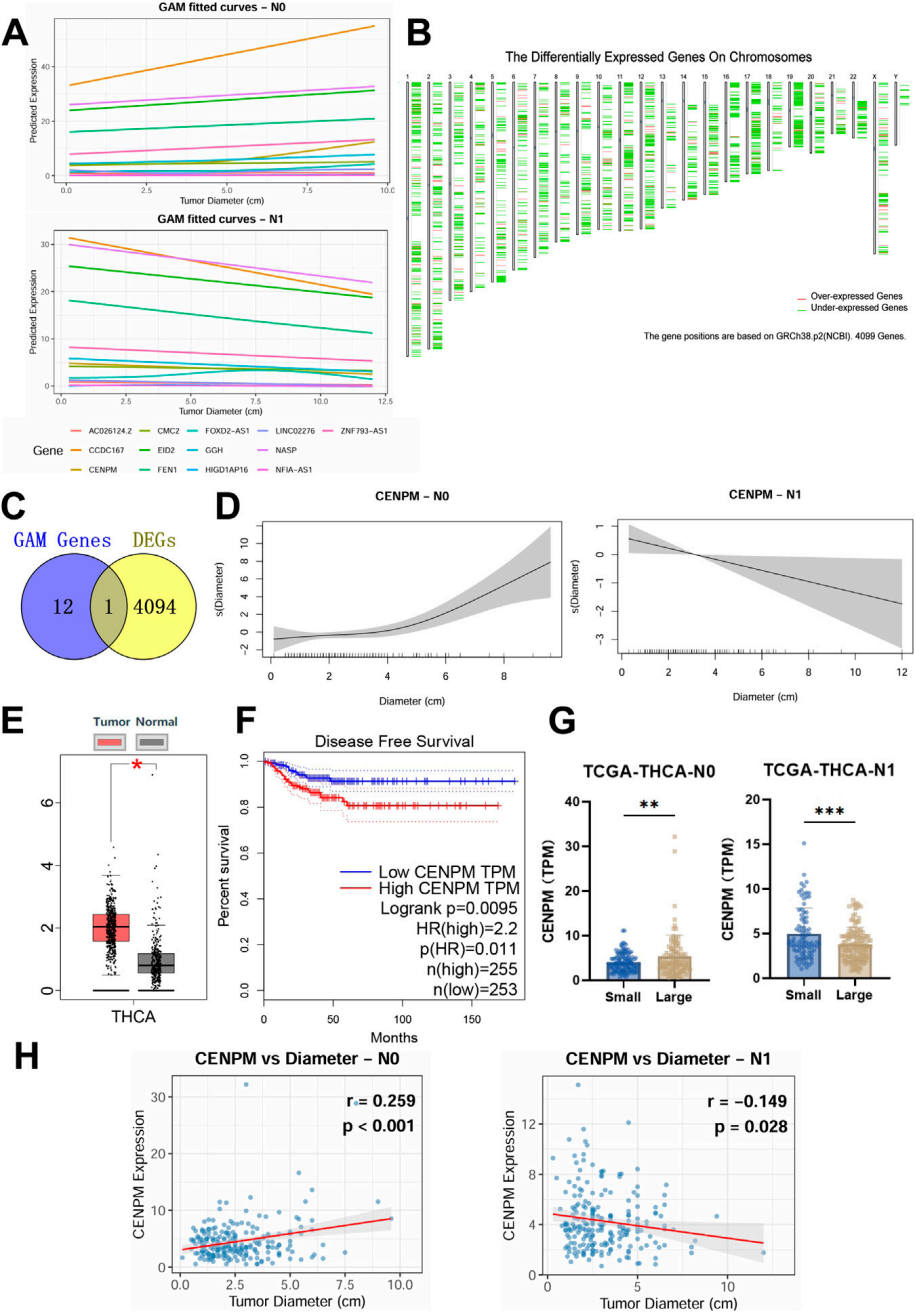
A GAM-based screening approach to identify potential markers of lymph node metastatic potential in THCA. **(A)** GAM fitted curves showing the predicted expression levels of candidate genes (including CENPM, AC026124.2, CMC2, FOXD2-AS1, LINC02276, ZNF793-AS1, CCDC167, EID2, GGH, NASP, FEN1, HIGD1AP16, and NFIA-AS1) as a function of tumor diameter in the N0 (left panel) and N1 (right panel) groups from the TCGA-THCA cohort. **(B)** Chromosomal distribution of DEGs between tumor and normal tissues in THCA from the GEPIA database. **(C)** Venn diagram showing the overlap between DEGs between tumor and normal tissues in THCA and genes identified by the GAM-based screening approach. **(D)** Smooth function plots (s(Diameter)) illustrating the nonlinear relationship between CENPM expression and tumor diameter in the N0 (left panel) and N1 (right panel) groups. **(E)** CENPM expression in THCA and normal thyroid tissues based on the GEPIA database. **(F)** DFS between CENPM-high and CENPM-low groups. **(G)** CENPM expression in N0 and N1 groups stratified by small versus large tumor diameter. **(H)** Correlation between tumor diameter and CENPM expression in the N0 and N1 groups. *P < 0.05, **P < 0.01, ***P < 0.001.

To identify genes whose expression patterns correlate with lymph node metastatic potential, we employed GAM to examine the relationship between gene expression and primary tumor diameter separately in lymph node-negative (N0, n = 220) and lymph node-positive (N1, n = 218) patients. Among the genes that exhibited an opposite association pattern (positive in N0 and negative in N1) with statistical significance in both groups (pattern 1), CENPM stood out as the most compelling candidate (Table 1; Figure 1A). We report both levels of correction simultaneously: (1) genome-wide Benjamini–Hochberg FDR correction, using the actual number of genes tested in each group as the correction baseline; and (2) internal FDR correction within the 13 selected genes. The fully corrected p-values are provided in Supplementary Table S1. Using the GEPIA2 database, we analyzed DEGs between tumor and normal tissues in the TCGA-THCA cohort (screening criteria: q < 0.05 and |logFC| > 1) (Supplementary Table S2; Figure 1B). This set of 4,099 DEGs was intersected with the 13 genes identified by the aforementioned GAM screening, and only CENPM was found to overlap (Figure 1C). This independent evidence lends support to the aberrant expression of CENPM in thyroid carcinoma and its biological significance that warrants further investigation, ultimately leading us to select CENPM for subsequent analysis.

**TABLE 1:** Genes with expression significantly associated with tumor diameter in N0 and/or N1 groups identified by generalized additive models (GAM).

Gene	delta_N0	p_GAM_N0	delta_N1	p_GAM_N1	edf_N0	edf_N1	dev_expl_N0	dev_expl_N1
CCDC167	21.69638872	0.037436849	−11.95980478	0.030729162	1.00153627	1.001975127	0.019754047	0.02149142
NASP	6.678634246	0.025934352	−8.028300069	0.00783596	1.000960704	1.001094184	0.022576797	0.032321399
FEN1	4.87977153	0.037891919	−6.92342955	0.003947554	1.000543957	1.07957011	0.019635768	0.041611195
EID2	7.305867741	0.015854488	−6.652752626	0.013874284	1.000986787	1.000058463	0.026433909	0.027703898
ZNF793-AS1	5.333176861	0.007125556	−2.917587974	0.038663277	1.0031357	1.000869269	0.032878307	0.019670497
GGH	3.305274026	0.039245988	−2.793284022	0.010934179	1.310220905	1.001352381	0.028486858	0.029656633
CENPM	8.704680634	8.49026E-05	−2.296436349	0.028281456	2.437764366	1.000087252	0.100752675	0.022078857
LINC02276	0.257412866	0.024253264	−1.180057402	0.031754264	2.753912798	1.000375636	0.053222958	0.021182207
CMC2	1.038700215	0.043068831	−0.917160432	0.04842775	1.000081759	1.000105257	0.01864295	0.0179146
AC026124.2	0.111910104	0.012813049	−0.619281578	0.012769269	2.916431657	1.000117659	0.061455765	0.028367499
FOXD2-AS1	2.978764786	0.001142067	−0.295412651	3.90374E-05	2.769519879	3.116144403	0.081958156	0.12152951
NFIA-AS1	0.132588656	0.04048189	−0.150695701	0.020057723	1.000383497	1.000105958	0.019126141	0.024780707
HIGD1AP16	0.319756174	0.004169174	−0.108063153	0.014746996	1.000056019	2.801942826	0.037039313	0.058925859

In the N0 group, CENPM expression increased significantly with tumor diameter (Δ = 8.70, p = 8.49 × 10^−5^, edf = 2.44, deviance explained = 10.1%), indicating a positive correlation. In contrast, in the N1 group, CENPM expression showed a significant negative correlation with diameter (Δ = −2.30, p = 0.028, edf = 1.00, deviance explained = 2.2%). The opposite direction of association was also visually confirmed by the fitted GAM curves (Figure 1D).

Based on the GEPIA database using the TCGA-THCA cohort, CENPM expression was significantly higher in tumor tissues compared to normal tissues (Figure 1E). Moreover, patients with high CENPM expression exhibited a significantly poorer disease-free survival (DFS) (log-rank p = 0.0095; HR = 2.2, p = 0.011; Figure 1F). Among patients without LNM (N0), CENPM expression was higher in larger tumors, whereas among patients with LNM (N1), CENPM expression was higher in smaller-diameter tumors (Figure 1G). Spearman correlation analysis revealed that in patients without LNM (N0), CENPM expression increased with tumor diameter—larger tumors corresponded to a higher risk of future LNM, i.e., greater metastatic potential. In contrast, in patients with established LNM (N1), where even a small tumor diameter already indicates a very high metastatic potential, CENPM expression decreased as tumor diameter increased (Figure 1H). These opposite correlation patterns across the two groups provide a rationale for further investigating CENPM as a molecular phenotype associated with differential biological behavior across nodal strata, rather than a simple correlate of tumor size.

## 13 Independent datasets and experimental validation

**FIGURE 2:**
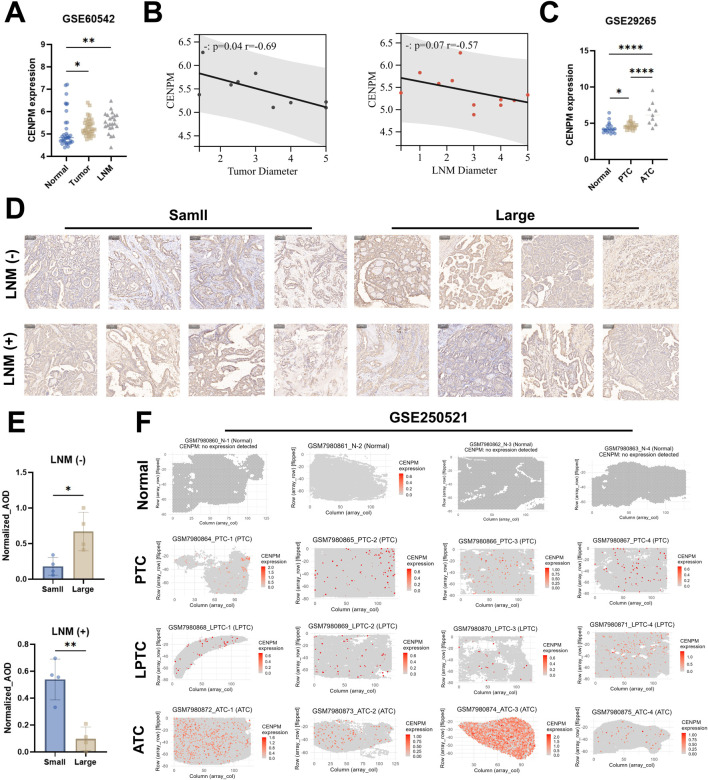
Independent validation and experimental verification of CENPM **(A)** CENPM expression in normal thyroid tissue, primary THCA, and metastatic lymph nodes in the GSE60542 dataset **(B)** Correlation analysis of CENPM expression with primary tumor diameter and metastatic lymph node diameter in the GSE60542 dataset **(C)** CENPM expression in normal thyroid, PTC, and ATC tissues in the GSE29265 dataset **(D)** Representative IHC images of CENPM in LNM− and LNM + THCA tissues subdivided by median tumor diameter (small vs. large). Scale bar, 100 µm **(E)** Comparison of AOD values across the indicated subgroups **(F)** CENPM expression levels in normal, PTC, LPTC, and ATC tissues from the GSE250521 spatial transcriptomics dataset. *P < 0.05, **P < 0.01, ****P < 0.0001.

In the GSE60542 cohort, CENPM expression was significantly upregulated in both cancer tissues and metastatic lymph nodes compared to normal thyroid tissues (Figure 2A). Among patients with LNM who had available tumor diameter data, correlation analysis revealed a asignificant negative correlation between CENPM expression and primary tumor diameter, as well as a negative correlation with the diameter of metastatic lymph nodes (Figure 2B). In an independent validation dataset (GSE29265), CENPM expression was higher in PTC tissues than in normal tissues. Moreover, CENPM expression was further elevated in ATC compared to PTC (Figure 2C). We further collected clinical samples and performed immunohistochemistry for additional validation. A total of 16 THCA samples were obtained, including 8 cases with pathologically confirmed LNM after lymph node dissection (LNM+), and 8 cases without LNM (LNM–) (Figure 2D). Patients in each group were dichotomized into smaller-diameter and larger-diameter subgroups based on the median tumor diameter. In the LNM–group, the AOD of CENPM was higher in the larger-diameter subgroup than in the smaller-diameter subgroup. Conversely, in the LNM + group, the larger-diameter subgroup exhibited a lower AOD of CENPM compared to the smaller-diameter subgroup. These findings were consistent with our previous observations (Figure 2E). In another independent validation dataset, spatial transcriptomics data from GSE250521 revealed the expression levels of CENPM in normal thyroid tissue, PTC, locally advanced thyroid cancer (LPTC), and ATC. Notably, CENPM expression was significantly elevated in all cancer samples compared to normal tissues (Figure 2F).

## 14 Single-cell transcriptomic analysis

**FIGURE 3:**
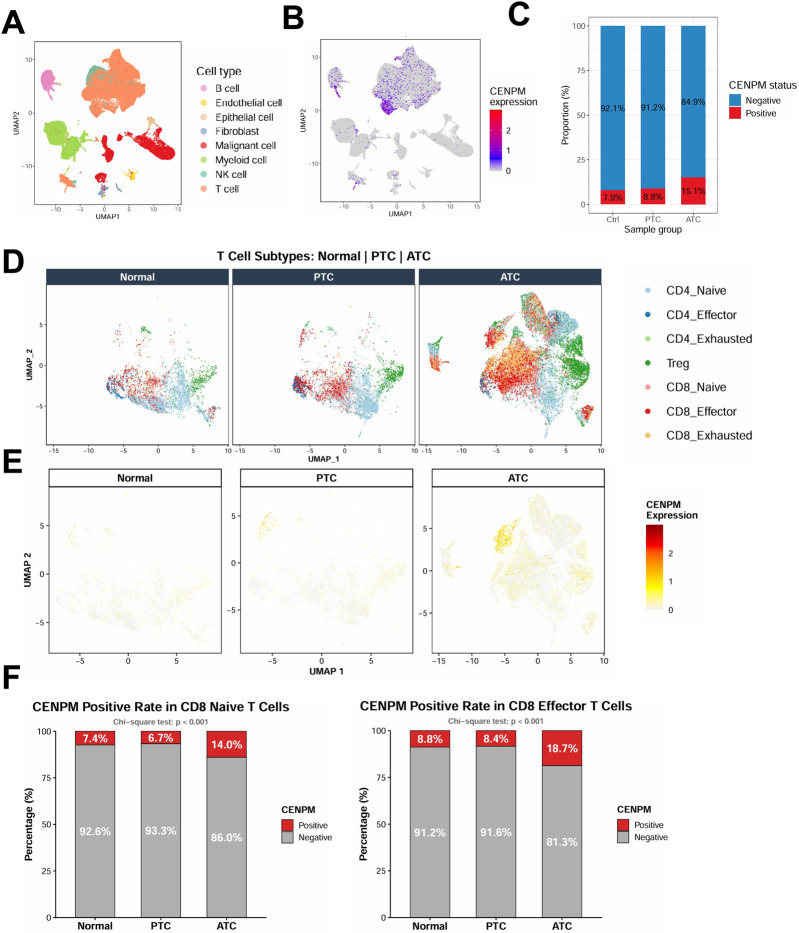
Single-cell analysis of CENPM in the GSE193581 dataset **(A)** UMAP visualization of major cell populations in thyroid tissues **(B)** CENPM expression levels across cell types, showing relatively high expression in T cells **(C)** Comparison of T cell infiltration proportions among normal, PTC, and ATC samples **(D)** UMAP plot of T cell subtypes identified across tissue origins **(E)** Overall proportion of CENPM-positive T cells in normal, PTC, and ATC **(F)** Percentages of CENPM-positive cells within CD8^+^ naive and CD8^+^ effector T cell subsets across normal, PTC, and ATC.

Single-cell analysis was performed using the GSE193581 dataset. UMAP dimensionality reduction revealed distinct cell populations, and CENPM average expression levels across cell types indicated that CENPM was relatively highly expressed in T cells (Figures 3A,B). The infiltration proportion of T cells was higher in ATC compared to normal thyroid tissue and PTC (Figure 3C). We further dissected distinct T-cell subtypes across the different tumor types (Figure 3D). The overall proportion of CENPM-positive T cells was elevated in ATC samples (Figure 3E). Notably, the proportions of CD8_Naive and CD8_Effector cells positive for CENPM (i.e., percentage of CENPM-expressing cells) were significantly increased in ATC relative to normal tissue and PTC (Figure 3F; Supplementary Figure S1). Previous studies have demonstrated an association between CENPM and cell cycle regulation. Accordingly, we further analyzed CENPM expression across different cell cycle phases in various T-cell subsets. In ATC, exhausted T cells in the G2/M phase—including both CD4_Exhausted and CD8_Exhausted subsets—exhibited significantly higher CENPM expression (Supplementary Figure S2). In PTC, CD8_Effector and regulatory T cells (Tregs) in the G2/M phase showed elevated CENPM expression. In normal samples, CD8_Effector cells in the G2/M phase also displayed higher CENPM expression levels.

## 15 Biological function exploration of CENPM

**FIGURE 4:**
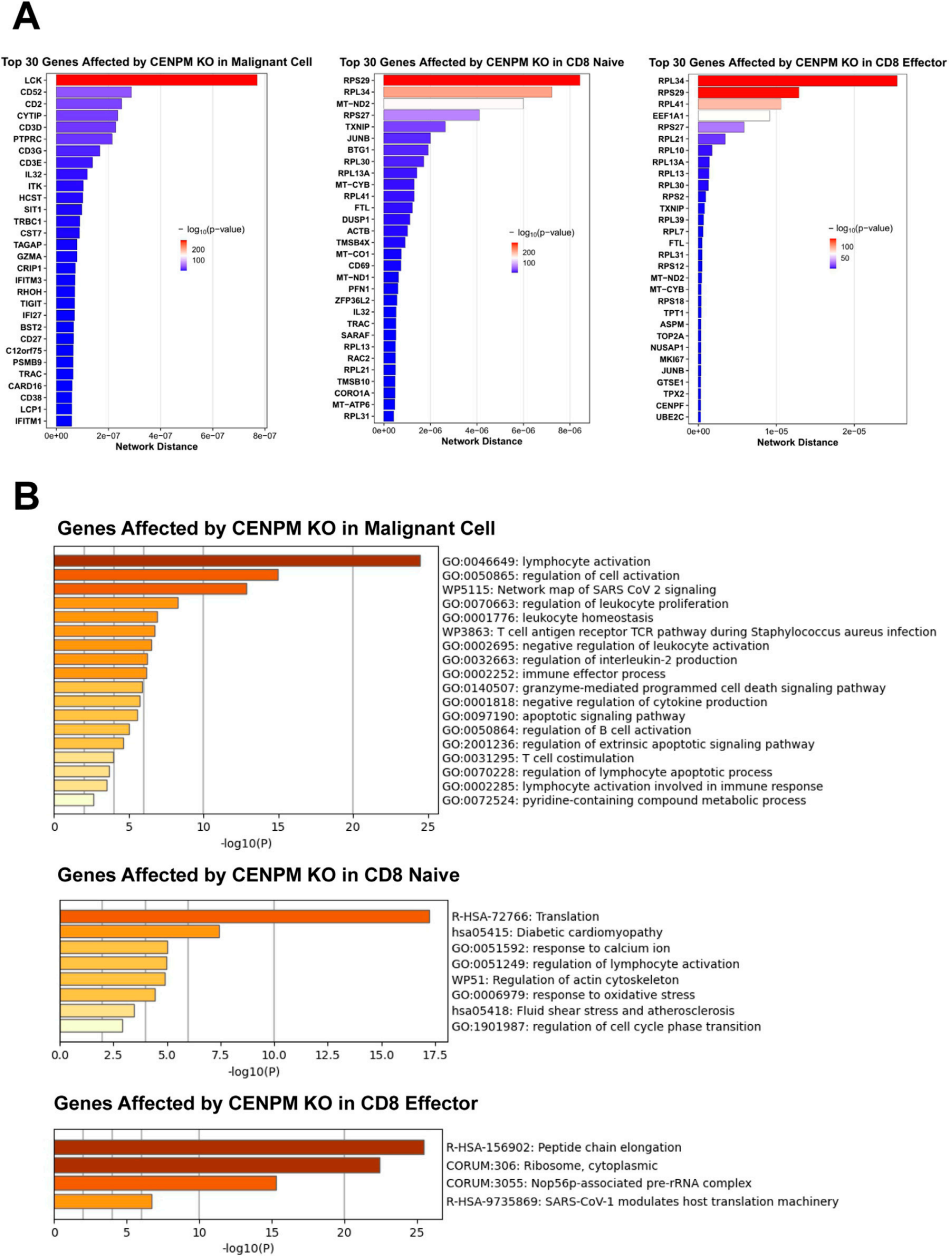
In silico knockout of CENPM by scTenifoldKnk **(A)** Top 30 genes most transcriptionally perturbed upon CENPM virtual knockout in malignant cells, CD8^+^ naive T cells, and CD8^+^ effector T cells, ranked by network distance **(B)** Metascape pathway enrichment analysis of genes significantly altered (P < 0.05) following CENPM ablation in each indicated cell type.

To elucidate the potential functional implications of CENPM in PTC and ATC, we performed an *in silico* gene knockout of CENPM using the scTenifoldKnk algorithm across malignant cells and the T-cell subpopulations exhibiting the most pronounced alterations in CENPM-positive proportions---specifically CD8 naive and CD8 effector T cells---from the GSE193581 cohort. Because scTenifoldKnk infers transcriptional consequences through gene co-expression network perturbation, its output reflects predicted associations rather than direct causal mechanisms. The top 30 genes exhibiting the greatest predicted transcriptional perturbation (network distance) following CENPM knockout were visualized for each cell type (Figure 4A). Subsequently, genes predicted to be significantly perturbed (p < 0.05) upon CENPM ablation in malignant cells, CD8 naive T cells, and CD8 effector T cells were subjected to Metascape pathway enrichment analysis, respectively (Figure 4B). In malignant cells, CENPM knockout was predicted to alter pathways associated with lymphocyte activation, regulation of leukocyte proliferation and homeostasis, T-cell antigen receptor (TCR) signaling, and immune effector processes. Notably, granzyme-mediated programmed cell death, apoptotic signaling pathways, and regulation of interleukin-2 production and cytokine secretion were also among the top predicted perturbations. In CD8 naive T cells, CENPM ablation was predicted to affect translation machinery, actin cytoskeleton organization, and oxidative stress responses, concomitant with regulation of lymphocyte activation and cell cycle phase transition. In CD8 effector T cells, CENPM knockout was predicted to alter ribosome biogenesis, peptide chain elongation, and cytoplasmic ribosome assembly, including Nop56p-associated pre-rRNA processing complexes.

## 16 Therapeutic exploration of CENPM

**FIGURE 5:**
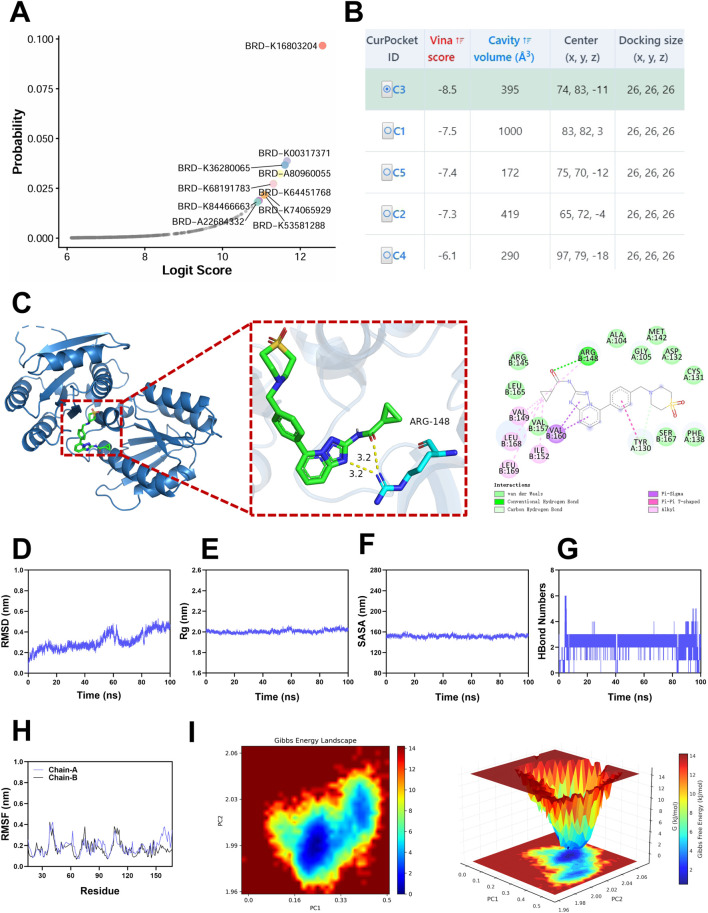
Drug repositioning, molecular docking, and molecular dynamics simulation of CENPM **(A)** DrugReflector screening identifies filgotinib as the top candidate **(B)** Docking poses in pockets C1–C5; optimal binding in C3 (Vina score = −8.5 kcal/mol) **(C)** Interaction diagram of filgotinib in pocket C3 **(D–I)** MD simulation analyses **(D)** RMSD, **(E)** Rg **(F)** SASA, **(G)** H-bond count **(H)** RMSF, and **(I)** free energy landscape.

To further assess the clinical translational value of our findings, we utilized the DrugReflector algorithm to systematically screen for small-molecule compounds capable of reversing the transcriptional signature associated with CENPM overexpression. BRD-K16803204 (Filgotinib, a selective Janus kinase [JAK] inhibitor) was identified as the top-ranked candidate, predicted to most effectively counteract the CENPM-driven transcriptional program (Figure 5A). We subsequently performed molecular docking of filgotinib against CENPM. Among the five predicted binding pockets (C1–C5), pocket C3 yielded the optimal conformation with a Vina score of −8.5 kcal/mol (Figure 5B), with intermolecular interactions detailed in Figure 5C. MD simulations were further conducted to assess dynamic stability (Figures 5D–I). RMSD analysis showed the system equilibrated after ∼85 ns, stabilizing around 0.46 nm (Figure 5D). The Rg profile remained steady without marked expansion or contraction (Figure 5E), and SASA revealed no appreciable change post-binding (Figure 5F), indicating a stable interface. H-bond analysis showed persistent intermolecular hydrogen bonds, fluctuating between 0 and 6 with an average of ∼2–3 across most frames (Figure 5G). RMSF profiling of both chains demonstrated predominantly low residual flexibility, with most residues below 0.2 nm (Figure 5H). FEL mapping identified a dominant low-energy basin at PC1 ≈ 0.25 nm and PC2 ≈ 1.98 nm (Figure 5I). Collectively, these data demonstrate that filgotinib forms a stable, high-affinity complex with CENPM, characterized by favorable H-bonding, low flexibility, and a thermodynamically favorable conformational basin, confirming robust target engagement.

## 17 Discussion

In this study, we introduced a novel analytical framework that leverages GAMs to dissect gene expression–tumor diameter relationships within distinct nodal strata, thereby transitioning from static anatomical staging to a dynamic, molecular assessment of lymph node metastatic potential. The core innovation of this GAM-based screening strategy lies in its abandonment of linear assumptions: by fitting expression–diameter curves with thin plate regression splines and requiring diametrically opposite association directions in the N0 and N1 cohorts, our pipeline intrinsically discriminates genes that reflect tumor-burden effects from those that genuinely mark metastatic propensity. Through this strategy, Through this strategy, CENPM was found to display opposite associations with tumor diameter between N0 and N1 cohorts in THCA. To our knowledge, this represents the first exploratory analysis linking CENPM expression patterns to nodal status and DFS in THCA.

CENPM, a constituent of the constitutive centromere-associated network, is canonically implicated in kinetochore assembly, chromosome congression, and cell-cycle fidelity (Liu et al., 2022c). Prior pan-cancer analyses have documented its oncogenic amplification across multiple solid malignancies; however, its specific relevance to THCA progression and nodal dissemination has remained entirely unexplored (Tang et al., 2026). Our TCGA–GAM analysis revealed a diametric association: CENPM expression increased with tumor diameter in the N0 cohort (Δ = 8.70, p = 8.49 × 10^−5^), whereas it exhibited a significant negative correlation in the N1 cohort (Δ = −2.30, p = 0.028). We interpret this paradox as evidence that CENPM marks intrinsic metastatic propensity rather than mere tumor bulk. In N0 disease, rising CENPM with enlarging diameter likely reflects an activated pre-metastatic transcriptional state during tumor expansion; conversely, in N1 disease, elevated CENPM in small-diameter tumors signifies aggressive biological behavior capable of early lymphatic dissemination independent of size. This interpretation was independently corroborated by bulk transcriptomic datasets (GSE60542, GSE29265), spatial transcriptomics (GSE250521), and quantitative IHC, wherein CENPM displayed a clear malignantness gradient (ATC > PTC > normal) and an inverse diameter–expression relationship in metastatic contexts.

Previous studies have shown that CENPM is aberrantly overexpressed in multiple malignant tumor types and is closely associated with tumor metastasis, including a tendency for regional LNM. In adrenocortical carcinoma, CENPM acts as a key driver gene by physically interacting with the immune checkpoint ligand FGL1, thereby promoting tumor cell proliferation, invasion, and liver metastasis (Zou et al., 2025). In pancreatic cancer, upregulation of CENPM enhances cell migration and invasion via the mTOR/p70S6K signaling pathway (Zheng et al., 2020). In ovarian cancer, CENPM overexpression correlates with proliferation, migration, and epithelial-mesenchymal transition, whereas CENPM knockdown inhibits tumor metastasis by activating the cGAS-STING pathway and inducing pyroptosis (Xie et al., 2024). Collectively, these findings suggest that CENPM plays a common pro-metastatic role across different cancer types, albeit through distinct molecular mechanisms.

Different from previous reports, our study, via single-cell sequencing analysis, revealed the association of CENPM with immune cell infiltration in THCA, with a specific focus on T cells. CENPM was predominantly expressed in T-cell lineages, with the highest positivity observed in CD8^+^ naive and CD8^+^ effector subsets, particularly within ATC. Emerging evidence indicates that the density, differentiation state, and spatial distribution of tumor-infiltrating CD8^+^ T cells profoundly influence nodal metastasis in solid tumors; yet the specific intracellular programs that govern T-cell competence in the THCA microenvironment remain incompletely defined.

Emerging evidence highlights the distinct and coordinated roles of naive and effector CD8^+^ T cell subsets in tumor LNM. Tumor-specific adaptive immunity is initiated within the tumor-draining lymph node (TdLN), where migratory type 1 conventional dendritic cells (cDC1 s) prime naive CD8^+^ T cells through cross-presentation of tumor antigens (Contassot et al., 2009; Chen and Mellman, 2013). However, as tumors progress, this critical priming site becomes increasingly compromised—CD8+ T cell dysfunction within the TdLN constitutes an early and fundamental hallmark of tumor immune escape (du Bois et al., 2021). At the transcriptional level, the T-box transcription factor Eomesodermin (Eomes) has emerged as a key regulator of CD8^+^ T cell effector function and LNM. Analysis of 88 human colorectal tumor specimens demonstrated a significant inverse correlation between Eomes expression and the presence of lymph node metastases (p < 0.02); mechanistically, Eomes was found to control cytolytic activity through direct regulation of perforin and IFN-γ production in tumor-infiltrating CD8^+^ T cells (Atreya et al., 2007; Rahim et al., 2023). In parallel, functional analysis of CD8^+^ T cells in human head and neck squamous cell carcinoma (HNSCC) revealed that progenitor exhausted CD8^+^ T cells (Tpex), which are abundant in uninvolved LNs and clonally related to terminally exhausted cells in the tumor, are functionally disrupted in metastatic LNs—thereby impairing the response to anti-PD-L1 immunotherapy (Atreya et al., 2007; Karlow et al., 2022). These findings converge on a pivotal therapeutic principle: the stem-like Tpex subset residing in the TdLN, rather than terminally differentiated intratumoral T cells, sustains durable CD8^+^ T cell infiltration into tumors and mediates the response to immune checkpoint blockade (Karlow et al., 2022). Collectively, the evidence underscores that the functional quality, differentiation trajectory, and spatial localization of CD8^+^ T cell subsets—not their mere abundance—dictate the balance between immune surveillance and metastatic escape in the lymph nodes, shifting the therapeutic focus toward strategies that restore T-cell functionality within the TdLN (du Bois et al., 2021).

Our observation that CENPM enriches specifically in CD8^+^ naive and effector populations—rather than exhausted subsets—within the most aggressive histological subtype suggests that CENPM may be associated with the proliferative states or transitional capacity. Further supporting this notion, CENPM expression was elevated in G2/M phase T cells, including exhausted subsets, in ATC, implying a link to cell-cycle progression that could influence the functional readiness of T cells confronting metastatic tumor cells. By potentially altering the activation trajectory and effector differentiation of CD8^+^ T cells, CENPM may reshape the immune microenvironment into a state permissive for lymphatic seeding.

To probe these mechanisms computationally, we employed scTenifoldKnk virtual knockout across malignant cells and the two T-cell subsets exhibiting the greatest CENPM-positive proportion shifts. Because scTenifoldKnk is built on gene co-expression networks, it predicts transcriptional consequences of gene perturbation rather than proving direct causal regulation. Moreover, CENPM is a core centromere-associated protein whose canonical function is cell cycle regulation and chromosome segregation; its knockout inevitably causes cell cycle arrest, which may indirectly affect all cell cycle-dependent processes, including lymphocyte activation and ribosome biogenesis. Bearing these limitations in mind, in malignant cells, CENPM knockout was predicted to alter pathways associated with lymphocyte activation, regulation of leukocyte proliferation and homeostasis, TCR signaling, and immune effector processes. Notably, predicted perturbations included granzyme-mediated programmed cell death and apoptotic signaling pathways, alongside regulation of IL-2 production and cytokine secretion (Grodzovski et al., 2011). These predicted changes may reflect downstream consequences of CENPM-mediated cell cycle disruption rather than direct regulation of immune microenvironment crosstalk or tumor-intrinsic survival signals. In CD8^+^ naive T cells, CENPM ablation was predicted to affect translation machinery, actin cytoskeleton organization, and oxidative stress responses, concomitant with regulation of lymphocyte activation and cell cycle phase transition. This may indicate that CENPM deficiency indirectly influences the structural integrity and synthetic capacity of naive CD8^+^ T cells through cell cycle arrest, rather than directly controlling their quiescent-to-activated transition potential (Ramanathan et al., 2009). In CD8^+^ effector T cells, CENPM knockout was predicted to alter ribosome biogenesis, peptide chain elongation, and cytoplasmic ribosome assembly, including pre-rRNA processing complexes. Previous studies have shown that these pathways potentially affected by CENPM are indispensable in CD8^+^ T cells for sustaining the high translational demands required for effector molecule synthesis and maintaining robust cytotoxic functionality (Tan et al., 2017; Araki et al., 2017). Collectively, these computational predictions suggest that CENPM deficiency may indirectly affect distinct cellular processes across compartments within the thyroid tumor microenvironment, but these inferences are based on correlation-based network propagation and cannot establish direct mechanistic control. This multi-faceted landscape explains why elevated CENPM can simultaneously signify an impending metastatic threat in N0 patients and an established, aggressive phenotype in N1 patients—it disarms anti-tumor immunity while fortifying tumor-intrinsic aggressiveness.

Building upon these functional insights, we pursued therapeutic translation through DrugReflector-based drug repositioning. Filgotinib, a selective JAK1 inhibitor currently approved for rheumatoid arthritis and ulcerative colitis (Dhillon and Keam, 2020). In this study, Filgotinib was identified as the top candidate predicted to reverse the CENPM-high transcriptional signature. Molecular docking revealed strong binding affinity (Vina score = −8.5 kcal/mol within pocket C3), and 100-ns MD simulations confirmed exceptional complex stability, sustained intermolecular hydrogen bonding (averaging 2–3 bonds), low residual flexibility (RMSF < 0.2 nm), and convergence toward a thermodynamically favorable conformational basin (PC1 ≈ 0.25 nm, PC2 ≈ 1.98 nm). The JAK–STAT signaling axis is centrally positioned in both tumor cell survival and immune cell activation (Xue et al., 2023). In malignant cells, JAK–STAT hyperactivation promotes proliferation and immune evasion; in T cells, it transduces cytokine signals essential for naive-to-effector differentiation, yet its persistent activation can drive T-cell exhaustion and functional impairment (Adachi et al., 2024). Given that our scTenifoldKnk analysis identified lymphocyte activation, cytokine regulation (including IL-2), and apoptotic resistance as top CENPM-dependent pathways, the convergence upon a JAK1 inhibitor is mechanistically coherent (Lensing and Jabbari, 2022). Filgotinib may simultaneously attenuate tumor-intrinsic CENPM-driven signaling and restore T-cell functional integrity by dampening aberrant JAK1 activity—suggesting a hypothetical dual-pronged mechanism that requires experimental validation. It is important to clarify that filgotinib’s selection by DrugReflector reflects its ability to reverse the CENPM-associated transcriptional signature, not a predicted direct interaction with CENPM protein. Filgotinib is a selective JAK1 inhibitor with no known structural or functional association with CENPM, a constitutive centromere protein. We therefore speculate that any therapeutic relevance of filgotinib in this context would operate through indirect, pathway-level mechanisms: by attenuating JAK–STAT signaling in tumor or immune cells, filgotinib may modulate the cellular environments or differentiation states in which CENPM-driven metastatic phenotypes emerge. The molecular docking and MD simulation presented here were intended as exploratory, hypothesis-generating analyses of a potential direct interaction, but they do not constitute evidence of stable binding or functional inhibition. Biochemical validation and cellular rescue experiments are imperative to test either the direct or indirect mechanism.

Several limitations warrant acknowledgment. The GAM-based screening was exploratory and hypothesis-driven rather than confirmatory. Although we applied Benjamini–Hochberg FDR correction at both the genome-wide and gene-set-internal levels, we acknowledge that stringent genome-wide correction may obscure modest but biologically relevant associations given the effect sizes observed in this study. We therefore report both correction frameworks for full transparency, and emphasize that all computational findings, including the prioritization of CENPM, require validation in independent cohorts and experimental models. First, the retrospective nature of TCGA and GEO datasets precludes definitive causal inference. Second, our IHC validation cohort was modest (n = 16), necessitating larger, multi-center prospective validation. Third, scTenifoldKnk and DrugReflector predictions are *in silico*. Specifically, scTenifoldKnk infers transcriptional consequences through gene co-expression network perturbation and cannot establish direct causal regulation. Because CENPM is a core centromere-associated protein whose canonical function is cell cycle regulation and chromosome segregation, its knockout inevitably induces cell cycle arrest, which may indirectly affect all cell cycle-dependent processes, including lymphocyte activation and ribosome biogenesis. Therefore, the pathway changes predicted by scTenifoldKnk may reflect secondary consequences of cell cycle disruption rather than direct CENPM regulation. Experimental validation, including cell cycle synchronization combined with CENPM knockdown/rescue assays, is required to distinguish direct regulatory roles from indirect effects. Pharmacological validation in THCA cell lines and patient-derived models is also imperative. Fourth, the proposed link between CENPM and JAK-STAT signaling, and the predicted therapeutic relevance of filgotinib in this context, are purely computational hypotheses. CENPM is not a known direct component of the JAK-STAT pathway. These predictions urgently require preliminary validation through *in vitro* cellular experiments, such as assessing JAK-STAT pathway activity (e.g., p-JAK1, p-STAT3 levels) in CENPM-modulated thyroid carcinoma cell lines. Fifth, the GAM-based screening was restricted to genes meeting predefined expression distribution criteria, which may have excluded other nonlinear metastatic drivers. Finally, the single-cell cohort (GSE193581), though comprehensive, may not capture the full inter-patient heterogeneity of THCA.

In conclusion, by integrating GAM-based transcriptomic screening with multi-modal validation and computational perturbation modeling, we establish CENPM as a candidate molecular phenotype for further investigation and a potential candidate for therapeutic exploration. Our findings lay the groundwork for prospective clinical trials evaluating CENPM-directed risk stratification and Filgotinib intervention in advanced THCA, thereby advancing LNM management from anatomical documentation to molecular risk assessment.

## 18 Conclusion

In conclusion, using a GAM-based screening approach, we identified CENPM as a potential marker of lymph node metastatic potential in THCA. Its expression showed opposite trends with tumor diameter in N0 and N1 patients, validated by independent datasets and immunohistochemistry. Single-cell analysis revealed CENPM enrichment in CD8^+^ naive and effector T cells, and virtual knockout predicted downstream transcriptional changes associated with lymphocyte activation, translation, and immune effector processes. Drug repositioning identified filgotinib as a candidate to reverse the CENPM-high signature, supported by stable docking and simulation. These findings position CENPM as a potential biomarker and therapeutic target for LNM in thyroid cancer, warranting further prospective validation.

## Data Availability

The data supporting the findings of this study are openly available in public repositories. Bulk RNA-sequencing and clinical data for thyroid carcinoma were obtained from The Cancer Genome Atlas (TCGA) via the Genomic Data Commons Data Portal (https://portal.gdc.cancer.gov/). Additional transcriptomic datasets analyzed in this study are accessible through the Gene Expression Omnibus (GEO) database (https://www.ncbi.nlm.nih.gov/geo/) under accession numbers GSE193581, GSE60542, GSE29265, and GSE250521. All other data generated or analyzed during this study are included in the article and its Supplementary Material.
