# Supplementary material for: CENPM as a biomarker and therapeutic target for lymph node metastasis in thyroid carcinoma
Source: Front Genet. 2026 Jul 22;17:1875148. doi: 10.3389/fgene.2026.1875148 (PMC13437095; doi:10.3389/fgene.2026.1875148)
Supplement: Supplementary file 1 [file Image1.pdf]

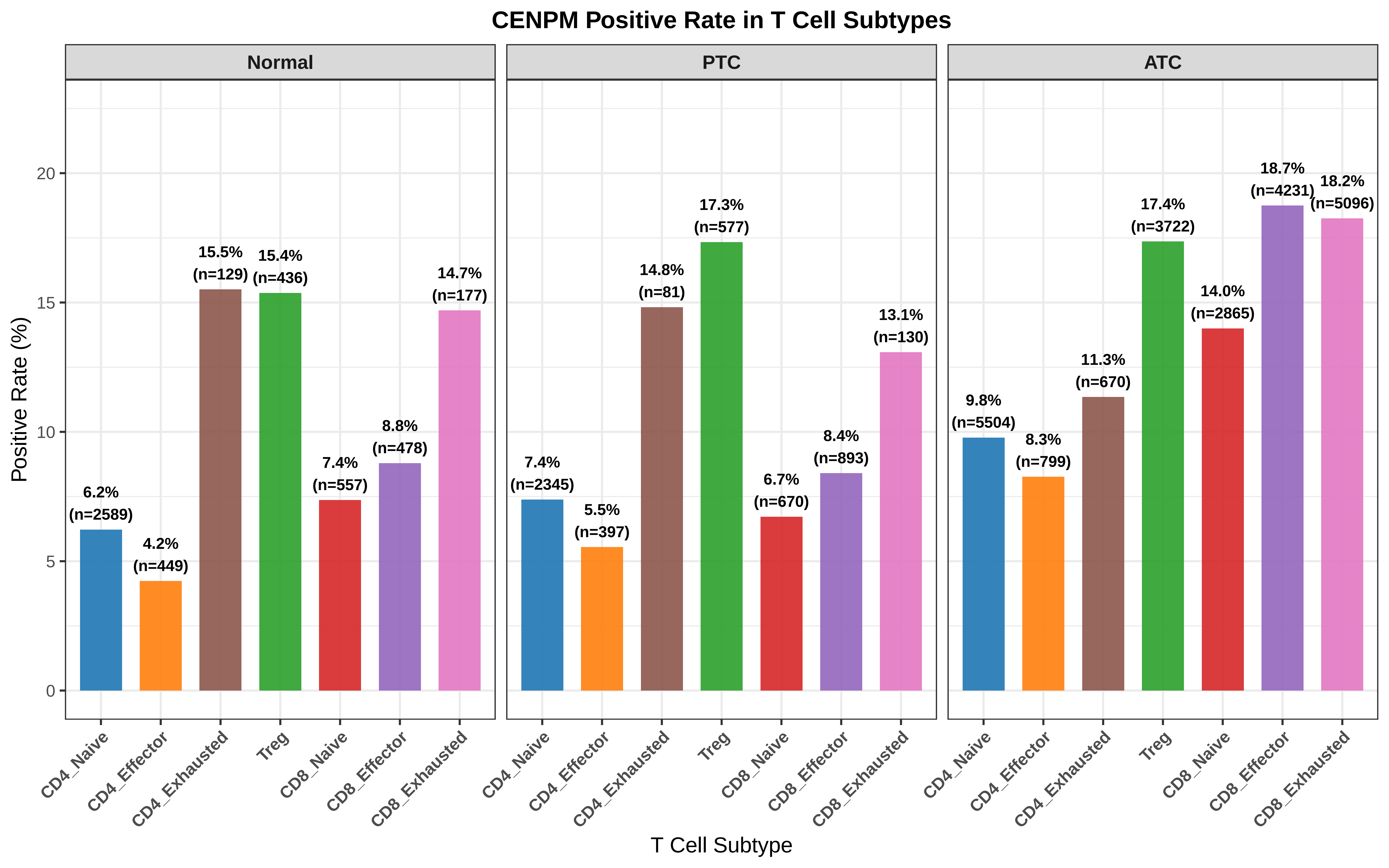

CENPM Positive Rate in T Cell Subtypes
Normal
PTC
ATC
Positive Rate (%)
0
5
10
15
20
6.2% (n=2589)
4.2% (n=449)
15.5% (n=129)
15.4% (n=436)
7.4% (n=557)
8.8% (n=478)
14.7% (n=177)
7.4% (n=2345)
5.5% (n=397)
14.8% (n=81)
17.3% (n=577)
6.7% (n=670)
8.4% (n=893)
13.1% (n=130)
9.8% (n=5504)
8.3% (n=799)
11.3% (n=670)
17.4% (n=3722)
14.0% (n=2865)
18.7% (n=4231)
18.2% (n=5096)
CD4_Naive
CD4_Effector
CD4_Exhausted
Treg
CD8_Naive
CD8_Effector
CD8_Exhausted
T Cell Subtype
