# Supplementary material for: CENPM as a biomarker and therapeutic target for lymph node metastasis in thyroid carcinoma
Source: Front Genet. 2026 Jul 22;17:1875148. doi: 10.3389/fgene.2026.1875148 (PMC13437095; doi:10.3389/fgene.2026.1875148)
Supplement: Supplementary file 2 [file Image2.pdf]

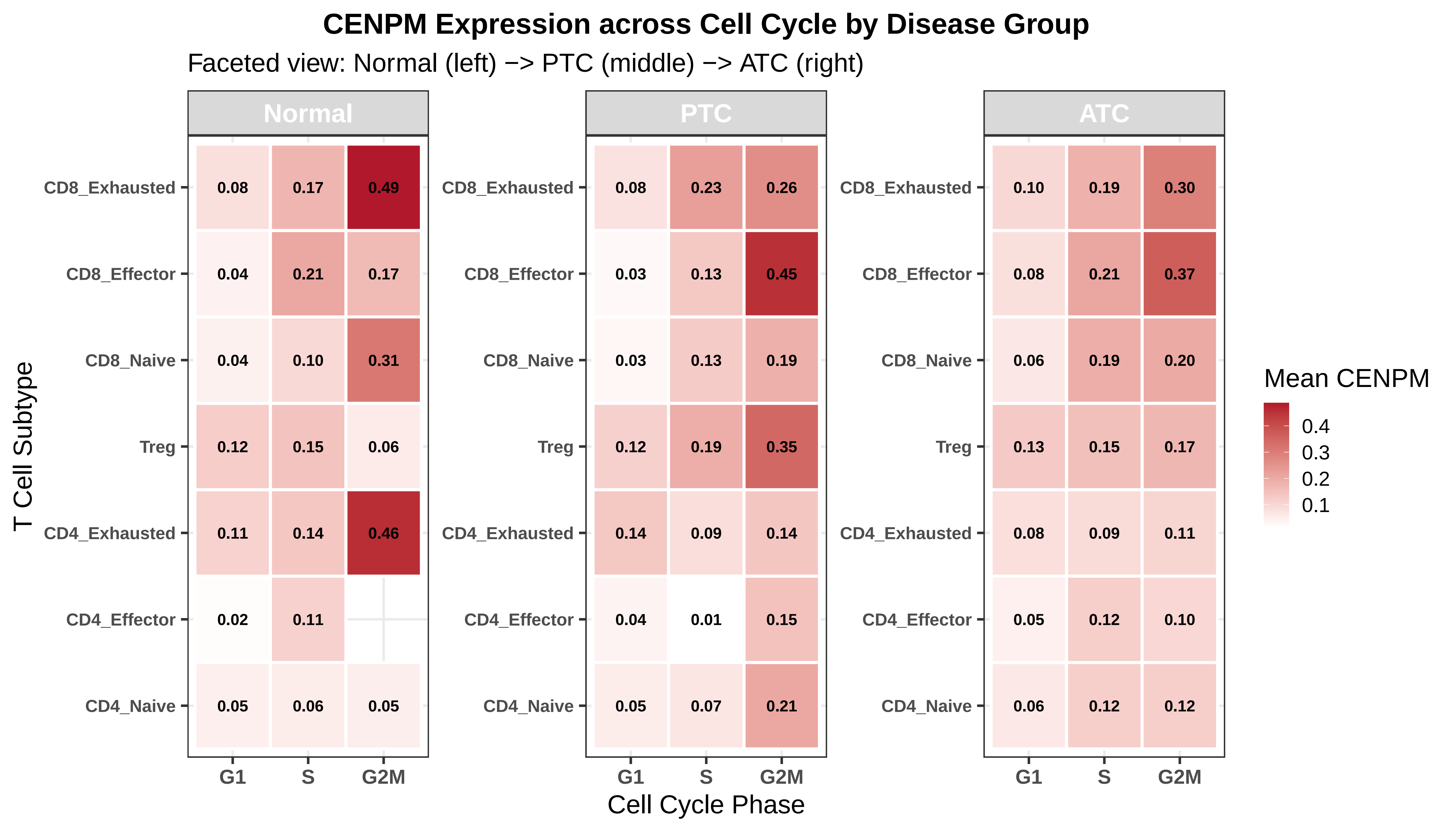

CENPM Expression across Cell Cycle by Disease Group
Faceted view: Normal (left) −> PTC (middle) −> ATC (right)
Normal
PTC
ATC
CD8_Exhausted
CD8_Effector
CD8_Naive
Treg
CD4_Exhausted
CD4_Effector
CD4_Naive
T Cell Subtype
0.08 0.17 0.49
0.04 0.21 0.17
0.04 0.10 0.31
0.12 0.15 0.06
0.11 0.14 0.46
0.02 0.11
0.05 0.06 0.05
0.08 0.23 0.26
0.03 0.13 0.45
0.03 0.13 0.19
0.12 0.19 0.35
0.14 0.09 0.14
0.04 0.01 0.15
0.05 0.07 0.21
0.10 0.19 0.30
0.08 0.21 0.37
0.06 0.19 0.20
0.13 0.15 0.17
0.08 0.09 0.11
0.05 0.12 0.10
0.06 0.12 0.12
G1
S
G2M
Cell Cycle Phase
Mean CENPM
0.4
0.3
0.2
0.1
